# Stress inducible proteinase inhibitor diversity in *Capsicum annuum*

**DOI:** 10.1186/1471-2229-12-217

**Published:** 2012-11-16

**Authors:** Manasi Mishra, Neha Mahajan, Vaijayanti A Tamhane, Mahesh J Kulkarni, Ian T Baldwin, Vidya S Gupta, Ashok P Giri

**Affiliations:** 1 Plant Molecular Biology Unit, Division of Biochemical Sciences, CSIR-National Chemical Laboratory, Dr. Homi Bhabha Road, Pune, MS, 411 008, India; 2 Department of Molecular Ecology, Max Planck Institute for Chemical Ecology, Jena, 07745, Germany; 3 Present address: Institute of Bioinformatics and Biotechnology, University of Pune, Pune, MS, 411 007, India

**Keywords:** Plant-insect interaction, Herbivory, Oral secretions, Pin-II type proteinase inhibitors, CanPI

## Abstract

**Background:**

Wound-inducible Pin-II Proteinase inhibitors (PIs) are one of the important plant serine PIs which have been studied extensively for their structural and functional diversity and relevance in plant defense against insect pests. To explore the functional specialization of an array of *Capsicum annuum* (L.) proteinase inhibitor (*CanPIs*) genes, we studied their expression, processing and tissue-specific distribution under steady-state and induced conditions. Inductions were performed by subjecting *C. annuum* leaves to various treatments, namely aphid infestation or mechanical wounding followed by treatment with either oral secretion (OS) of *Helicoverpa armigera* or water.

**Results:**

The elicitation treatments regulated the accumulation of *CanPIs* corresponding to 4-, 3-, and 2-inhibitory repeat domains (IRDs). Fourty seven different *CanPI* genes composed of 28 unique IRDs were identified in total along with those reported earlier. The *CanPI* gene pool either from uninduced or induced leaves was dominated by 3-IRD PIs and trypsin inhibitory domains. Also a major contribution by 4-IRD *CanPI* genes possessing trypsin and chymotrypsin inhibitor domains was specifically revealed in wounded leaves treated with OS. Wounding displayed the highest number of unique *CanPIs* while wounding with OS treatment resulted in the high accumulation of specifically *CanPI-4*, *-7* and −*10*. Characterization of the PI protein activity through two dimensional gel electrophoresis revealed tissue and induction specific patterns. Consistent with transcript abundance, wound plus OS or water treated *C. annuum* leaves exhibited significantly higher PI activity and isoform diversity contributed by 3- and 4-IRD *CanPIs*. *CanPI* accumulation and activity was weakly elicited by aphid infestation yet resulted in the higher expression of *CanPI*-26, *-41* and −*43*.

**Conclusions:**

Plants can differentially perceive various kinds of insect attacks and respond appropriately through activating plant defenses including regulation of PIs at transcriptional and post-translational levels. Based on the differentially elicited *CanPI* accumulation patterns, it is intriguing to speculate that generating sequence diversity in the form of multi-IRD PIs is a part of elaborative plant defense strategy to obtain a diverse pool of functional units to confine insect attack.

## Background

Plants have evolved elaborate defense strategies composed of constitutive and inducible responses in order to cope with herbivore challenges. The induced defenses commence only when herbivore-derived signals are perceived by the plants. A wide array of studies has reported the induction of direct and indirect plant defenses in response to herbivory and other biotic stresses [1-4]. Insect damage, mechanical wounding and/or elicitors in insect oral secretions (OS), such as fatty acid amino acid conjugates, volicitin, inceptins, caeliferins, and glucose oxidase, stimulate the local and systemic release of signaling intermediates like systemin and/or jasmonic acid; these then amplify the defense cascade throughout the plant [5-7]. Though the major consequence of herbivory is wounding, plants' responses to insect feeding are more complex due to the elicitors present in insect OS [8]. Defense responses entail the regulated activation of plant defense genes and the suppression of growth-related genes [8,9]. As a result, defensive metabolites and/or proteins accumulate in plants within the local tissues damaged by herbivores as well as systemically in undamaged tissues.

The accumulation of trypsin and chymotrypsin-like proteinase inhibitors (PIs) throughout the aerial tissues of tomato and potato plants was demonstrated to be a direct consequence of insect-mediated damage or mechanical wounding [10]. Thus, serine PIs represent one of the best examples of locally and systemically induced responses in Solanaceous plants [11-16]. The constitutive expression of PIs, which has been reported to occur in storage organs and the reproductive tissues of plants, may fulfill anti-insecticidal as well as other endogenous functions *in planta*[4,13,16-18].

Most Solanaceae members contain the multi-gene family encoding Pin-II type PIs [4,16,19], which possesses considerable sequence diversity resulting from variations in tandem sequence repeats, domain duplications and circularly permuted domain organizations [20]. A distinct feature of these PIs is the presence of tandem repeats of a 50-amino-acid polypeptide called inhibitory repeat domain (IRD), which can vary from 1 to 8 with inter-connecting linker peptides. Each IRD contains 8 conserved cysteines (Cys) along with a reactive site for targeting a serine protease. Gene duplication events have resulted in the evolution of the multi-domain Pin-II family of PIs with structurally and functionally divergent IRDs [21]. Horn et al. [22] isolated a set of IRDs resulting from the differential proteolysis at the linker peptide separating the subunits of a 7-domain precursor from methyl-jasmonate-elicited *N. attenuata* leaves. The sequence variability in the multi-gene family of Pin-II PI proteins, their regulated expression and their post-translational processing are together responsible for generating a PI cocktail effective in defense and/or endogenous function [4,16,23].

Several different PI proteins and genes with 1- to 4-IRDs have been identified and characterized from *C. annuum* (CanPIs) tissues [16,24-29]. There was substantial variability in the induced expression of *CanPIs* upon aphid infestation, virus infection, chewing by insects and mechanical wounding. The abundance of transcripts did not always result in higher CanPI proteins, though they were well correlated in lepidopteran-infested *C. annuum* leaves. Furthermore, these studies indicated that many *CanPIs* are expressed simultaneously, but the significance of such PI expression diversity in *C. annuum* remains unclear.

In order to examine the potential functional specificities of the various isoforms of CanPI in *C. annuum*, we addressed the following questions: (i) Does elicitation increase PI isoform diversity? (ii) How specialized is the induction response to a particular treatment? Following experimental inductions of *C. annuum* leaves, we investigated the diversity in CanPI transcript and protein profiles. Sequencing revealed 24 novel *CanPI* transcripts, increasing the total known to 47. Selective analysis of PI activity in proteomes using 1D and 2D electrophoresis followed by mass spectrometry revealed local and systemic responses in PI activity.

## Results

### Differential regulation of *CanPIs* upon induction

**Figure 1:**
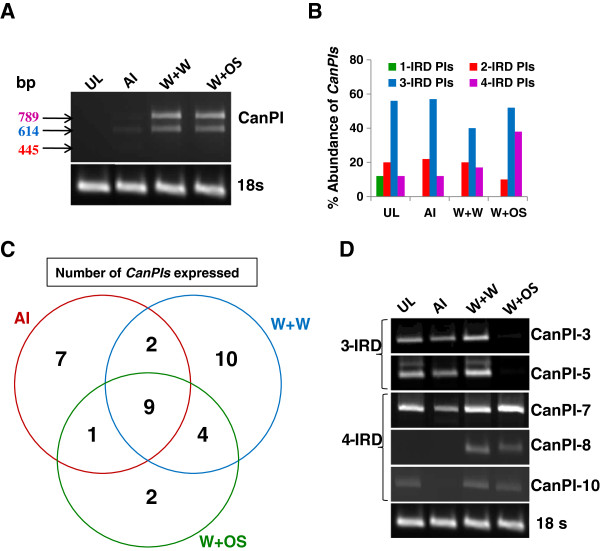
**Expression analysis of *CanPIs *under induced conditions.** [**A**] RT-PCR amplification (25 cycles) of mRNA from uninduced (UL) and systemic leaf tissues of induced *C. annuum* infested with aphids (AI), or wounded and treated with water (W+W) or oral secretions (W + OS). Details of primers used are given in Additional file 1: Table S1. cDNA templates were normalized based on *18S rRNA* amplification. [**B**] Abundance of 1-, 2-, 3- and 4-IRD *CanPIs* upon each induction treatment to *C. annuum* leaves. Abundance represents the frequency of a specific type of *CanPI* in the total clones sequenced per induction. The up-regulation of 4-IRD *CanPIs* in W+OS is evident. [**C**] Comparison of *CanPI* expression patterns in *C. annuum* leaves after different inductions. The Venn diagram presents the number of *CanPIs*, common or differentially expressed upon each type of induction; AI, W+W or W+OS. W+W showed the highest number of expressed *CanPIs*. [**D**] Expression analysis of selected *CanPIs* in un-induced and systemic leaf tissue of induced *C. annuum*. The details of gene-specific primers are given in Additional file 1: Table S1.

The amplification of cDNA derived from uninduced and induced leaves with *CanPI* gene-specific oligonucleotides yielded transcripts of 789, 614, 445 and 267 bp, representing 4-, 3-, 2- and 1-IRD *CanPIs,* respectively. Semi-quantitative analysis revealed differential *CanPI* expression in uninduced and induced leaves (Figure 1A). In comparison to *CanPIs* with 2-IRDs, those with 4- and 3-IRDs showed higher abundance in wounded leaves treated with water or OS (Figure 1A). In aphid-infested leaves, while the overall expression of *CanPIs* was low, 3-IRD transcripts were prominent. The amplified transcripts were cloned and 60 representative clones from each treatment (25 in case of uninduced) were sequenced to confirm the identities of the *CanPIs.* This analysis detected novel 4-, 3- and 2-IRD subtypes based on variations in amino acid composition. Based on this analysis, in addition to the 23 *CanPIs* previously reported [16,26,27], 24 novel *CanPIs* were identified including seven 2-IRD, fourteen 3-IRD, and three 4-IRD *CanPIs* (Table 1). 1-IRD *CanPIs* identified in uninduced leaves were not detected under any of the induction treatments. Details regarding the treatment-specific representation of *CanPIs* and their IRD composition are summarized in Table 1. The frequency of occurrence of individual *CanPIs* out of the 60 clones sequenced per treatment was analyzed and is referred to as abundance in leaf under a particular treatment (Table 1).

**Table 1:** **IRD composition and induction patterns of *C. annuum *Pin-II PI genes**

**Name**	**SP**	**1-IRD**	**2-IRD**	**3-IRD**	**4-IRD**	**Abundance in leaf**			
						**UL**	**AI**	**W+W**	**W+S**
***CanPI-*1**	5	4	5	10		2	1		
***CanPI-*2**	2	1	16	13					1
***CanPI-*3**	1	1	1	17		3	3	2	1
***CanPI-*4**	1	1	25	17			3	1	11
***CanPI-*5**	4	1	1	17		6	1	3	3
***CanPI-*6**		2	25	17	p	ND	ND	ND	ND
***CanPI-*7**	5	4	14	5	10	1	5	1	12
***CanPI-*8**	5	4	14	3	10			2	3
***CanPI-*9**	1	1	25	5	10	ND	ND	ND	ND
***CanPI-*10**	5	4	14	5	8	2		3	6
***CanPI-*11**	1	4	14	5	10			3	1
***CanPI-*12**		1	1	11	p	ND	ND	ND	ND
***CanPI-*13**	1	17				1			
***CanPI-*14**	1	6				ND	ND	ND	ND
***CanPI-*15**	5	7				2			
***CanPI-*16**	1	1	17			1			
***CanPI-*17**	1	12	17			1	1	1	1
***CanPI-*18**	1	25	17			1			
***CanPI-*19**	1	1	25			ND	ND	ND	ND
***CanPI-*20**	1	25	17						3
***CanPI-*21**	5	4	17			1			
***CanPI-*22**	5	4	9				1	2	2
***CanPI-*23**	3	25	17			1			
***CanPI-24***	1	1	18				5	4	
***CanPI-25***	3	1	18				2		
***CanPI-26***	5	4	10				4	1	
***CanPI-27***	5	23	18					1	
***CanPI-28***	8	22	18					1	
***CanPI-29***	6	21	18					1	
***CanPI-30***	7	1	17					1	
***CanPI-31***	1	1	12	27	17		1		1
***CanPI-32***	5	4	14	5	10		1		
***CanPI-33***	8	24	1	12	17			1	
***CanPI-34***	1	1	12	17				1	
***CanPI-35***	8	1	12	17				1	
***CanPI-36***	8	5	37	18				1	
***CanPI-37***	8	33	37	48		1		2	3
***CanPI-38***	9	5	40	17				1	
***CanPI-39***	8	5	12	17			1		
***CanPI-40***	5	5	37	18			1		
***CanPI-41***	8	5	37	18			10	4	5
***CanPI-42***	5	4	1	17			1		
***CanPI-43***	1	1	12	17		2	11	4	4
***CanPI-44***	1	1	12	17			1		
***CanPI-45***	10	1	12	18				2	
***CanPI-46***	1	4	37	18			1	1	1
***CanPI-47***	1	1	1	55				1	2
**p Partial sequence**									
**ND Not detected**									

The general architecture of a Pin-II PI gene is displayed on the top of the table. A typical *CanPI* gene consists of a signal peptide (SP) followed by 1, 2, 3 or 4 IRDs interconnected by linkers. The *CanPI* genes are listed as follows: 1-IRD type; 2-IRD type; 3-IRD type and 4-IRD type. SP denotes signal peptide and the numbers indicate any of the 10 different sequence variants. The position of IRDs is indicated at the top of the columns and the numbers indicate the occurrence of any of the 28 different IRDs at given position. *CanPI-1* and −*2* are from Kim et al., [2001] and Shin et al., [2001], respectively while *CanPI-3* to −*23* are from Tamhane et al., [2009]. *CanPI-24* to −*47* were identified in the present work. The frequency of occurrence of individual *CanPIs* in leaves from 60 clones analyzed from each induction treatment is given on the right side of the table. Nucleotide sequences of *CanPI-24* to −*47* have been submitted to NCBI (Accession numbers JX106474 to JX106497).

Variation in the abundance of *CanPIs* was apparent (Figure 1B), with 3-IRD *CanPIs* being highest (from 40 to 60%) either in uninduced or induced leaves. The abundance of 4-IRD *CanPIs* was increased in leaves subjected to wounding and treated with OS (38%), compared to aphid-infested (12%) leaves or in wounded leaves treated with water (17%). The proportion of 2-IRD *CanPIs* ranged from the lowest (10%) in wounded leaves treated with OS to the highest (20%) in aphid infested and wounded leaves treated with water. The differential expression of the various subtypes of *CanPIs* (with respect to their IRD composition; see Table 1) resulted in an induction-specific *CanPI* profile. *CanPI-4, -7, -10, -24,-41* and −*43* showed the highest representation in leaves across all induction treatments (Table 1). In wounded leaves treated with OS, *CanPI-4 and −7* showed the highest frequency, whereas *CanPI-41 and −43* were represented most highly in aphid-infested leaves. Wounded leaves treated with water showed the highest number of expressed *CanPIs* (25) as well as a wide representation of several unique *CanPIs* (10 in number) in low frequencies (Table 1, Figure 1C). Responses to wounding with OS appear more specialized as suggested by the expression of a few CanPIs (16) in high frequencies (Table 1, Figure 1C). Both the treatments share more *CanPIs* (3- and 4-IRD type) possibly due to the standardization of the amount of wounding between these two treatments. Aphid-infested leaves also accumulated transcripts of 7 unique *CanPIs* representing a diverse array of PIs. Only transcripts specific for *CanPI-3*, *-5*, *-7*, *-8* and −*10* could be analyzed from these tissues. *CanPI-7* (4-IRD) showed constitutive expression in uninduced as well as induced leaves, while *CanPI-3* and −*5* (3-IRD PIs) showed low accumulation levels in wounded leaves treated with OS (Figure 1D). *CanPI-8* and −*10* (4-IRD PIs) were differentially expressed in wounded leaves treated with water or OS, and completely absent in aphid-infested leaf tissues (Figure 1D).

The Protein Prowler predictor [30] revealed ER signal peptides in all *CanPIs*. The ER signal peptide (SP) sequences of 25 aa at the N-terminal of *CanPIs,* which showed ten variants, were named SP-1 to SP-10 (Additional file 2: Figure S1).

**Figure 2:**
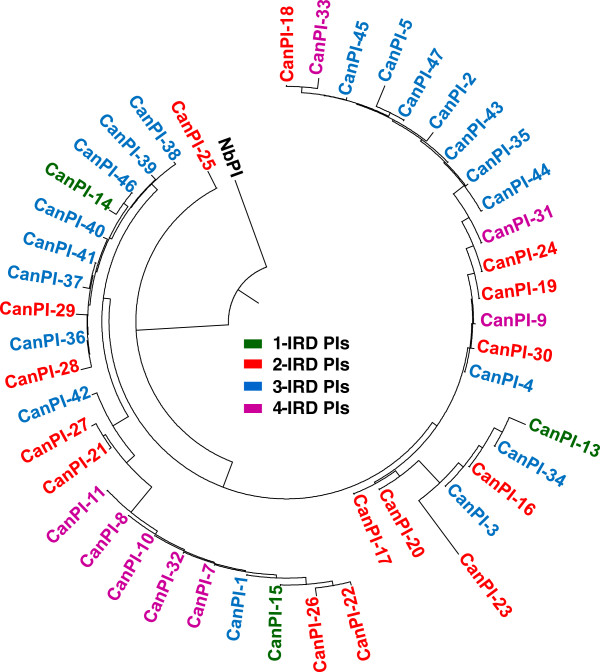
**Dendrogram based on aa sequence of *CanPIs *.** Dendrogram of *CanPIs* based on deduced amino acid sequences of full-length genes of 1- to 4-IRDs isolated from C. *annuum*. Pin-II type PI from *N. benthamiana* (*NbPI*, NCBI: ABA42892) is used as an out-group. Details of the *CanPIs* (1 to 47), composition, number of IRDs, tissue-wise representation and color coding are given in Table 1. Dendrogram was constructed using Lasergene software.

To investigate the interrelationships and groupings within *CanPIs* and with out-groups, phylogenetic analysis was carried out. *CanPIs* formed a distinct cluster from full-length Pin-II PI (4-IRD) from *Nicotiana benthamiana* used as an out-group. The dendrogram revealed clustering based on the identical component IRDs in *CanPIs* (Figure 2). Distinct clusters of 4- and 3-IRD PIs were evident with some intermixing for e.g. CanPI-31, -33, -1 and −4. *CanPIs* showing more aa sequence similarity associated close to each other for e.g. CanPI-8, -10 and −11, CanPI-37, -40 and −41, CanPI-13, -16 and −34.

**Figure 3:**
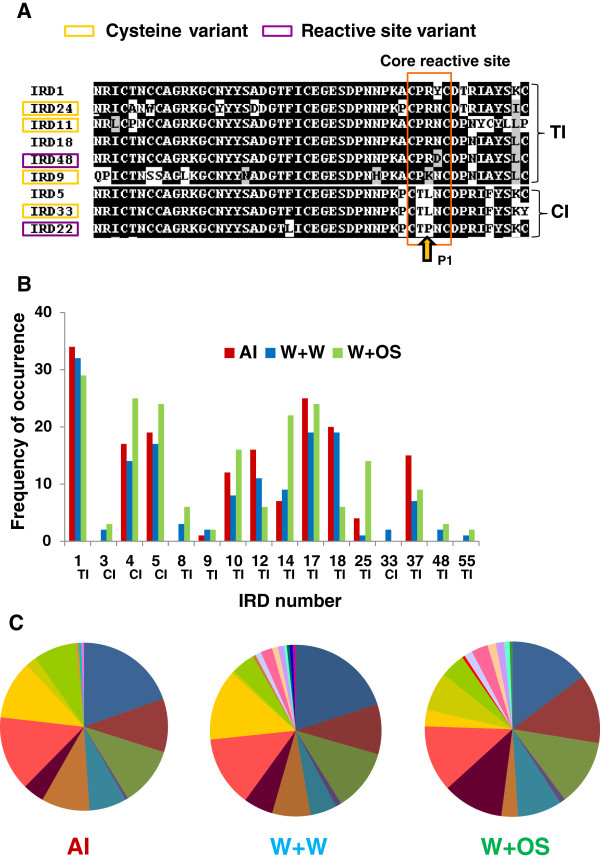
**Induction specific patterns of IRD expression.** [**A**] Multiple sequence alignment of deduced aa sequences of selected IRD variants among all *CanPI* genes. IRD1/IRD18 represents a typical trypsin inhibitory domain (TI) and IRD5 represents a typical chymotrypsin inhibitory domain (CI). The selected IRDs showing variation in the number of cysteines or active site residues are marked by yellow and purple boxes, respectively. The reactive site residue P1 is marked by an arrow. The presence of Lys (K) or Arg (R) at the P1 site indicates trypsin inhibitory site, and Leu (L) indicates chymotrypsin inhibitory site. The core reactive site is marked by an orange box. [**B**] The frequency of occurrence for individual IRDs per treatment. IRDs occurring only once were excluded from this graph. The highest representation of IRDs 1, 4, 5 and 17 across all treatments is apparent. [**C**] Pie charts represent the distribution of IRDs under each induction. A diverse array of unique IRDs is observed in wounded leaves treated with water or OS.

The deduced amino acid sequences of *CanPIs* (47 in total) consisted of 28 unique IRDs; of these, 7 had chymotrypsin inhibitory (CI) reactive sites and 21 had trypsin inhibitory (TI) reactive sites. The multiple sequence alignment of IRDs revealed variation mostly within reactive site loops or towards the C-terminal ends (Additional file 3: Figure S2). Five TI IRDs possess ‘Lys’ while 16 had ‘Arg’ at the P1 position of the reactive site. CI IRDs had ‘Leu’ at P1, except IRD 22 which had ‘Pro’. IRD 48 (TI) had active site variation with ‘Asn’ being replaced with ‘Asp’ (Figure 3A). Another crucial difference was variation in the number and positioning of cysteine residues per IRD. Four such cysteine variants, namely IRD 9, 11 and 24 were TIs, and IRD 33 was a CI (Figure 3A).

The relative abundance of IRDs that resulted from various types of inductions was analyzed (Figure 3B). Representation of IRDs 1, 4, 5, 10, 12, 14, 17, 18, 25 and 37 was high in induced tissues and exhibited treatment specificity. IRDs 1 and 17 were highly represented under all treatments while IRDs 12, 18 and 37, which are TIs, showed high frequency in aphid-infested leaves and wounded leaves treated with water (Figure 3B). The abundance of IRDs 4 and 5, which are CIs, and IRDs 10, 14 and 25, which are TIs, was distinct in wounded leaves treated with OS (Figure 3B). Most of the remaining diversity of IRDs is contributed by wounded leaves treated with water or OS (Figure 3C).

### Wounding and insect damage results in quantitative and qualitative changes in CanPI proteins

**Figure 4:**
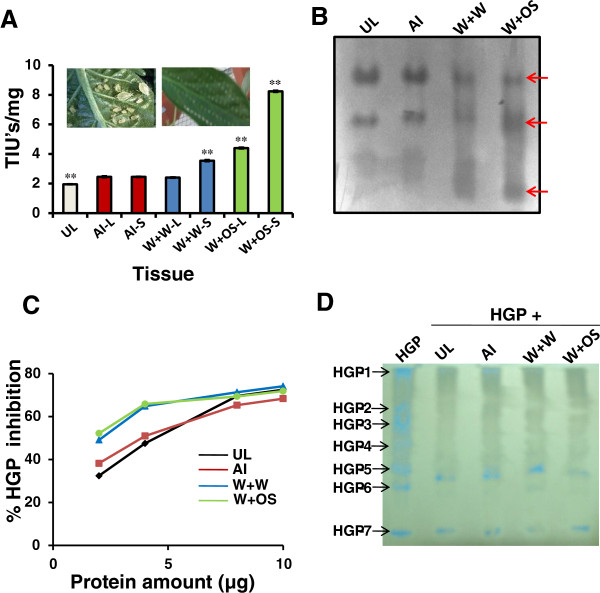
**PI activity in *C. annuum *uninduced and induced leaves.** [**A**] The accumulation of trypsin inhibitory activity in leaves infested with aphids (AI), wounded and treated with water (W+W) or oral secretions (W+OS). Local (L) and systemic (S) tissues corresponding to the respective induction are represented. The W+OS systemic tissue shows statistically significant higher trypsin inhibitory units (TIUs) (Tukey’s *t*-test at *p*<0.01). [**B**]* In-gel TI profiles of induced *C. annuum* leaves. 3 TI bands were visualized in W+W and W+OS treated leaves while only 2 in uninduced and aphid infested leaves. [**C**]* The percentage inhibition of HGP by PIs from leaf extracts estimated using azocasein as substrate. [**D**]* The inhibition of HGP isoforms by leaf extracts. Equal HGPI units of each of the extracts were incubated with HGP for 1 h at 25 ^0^C and visualized for residual protease activity. *The represented figures of induced tissues belong to systemic tissues of the particular induction treatment. Similar observations for local tissues of induction treatments.

*C. annuum* leaves were found to have increased PI activity upon induction (Figure 4A). Significantly higher level of PI activity was evident in the systemic leaves of wounding treated with water induction and both, the local and systemic leaves of wounding treated with OS induction compared to similar leaves from unwounded control plants (Figure 4A). The PI activity in aphid-infested leaves was higher than in uninduced leaves; however, it was 1.5- and 3.5-fold less than in wounded leaves treated with water or with OS, respectively. Induced levels of PI activity ranged from 2-fold in leaves wounded and treated with water to 4-fold in leaves wounded and treated with OS.

A differential pattern of PI isoform induction was observed in *C. annuum* leaves in response to various treatments (Figure 4B). Three prominent CanPI activity bands were detected in leaves that were wounded and treated with OS or with water, while only two PI isoforms could be detected in case of aphid infested and uninduced leaves. This difference indicated induced qualitative diversity in the CanPIs that resulted from these two treatments. Extracts from leaves wounded and treated with water or OS attained an early saturation of *H. armigera* gut protease (HGP) inhibition (70%) unlike aphid-infested and uninduced tissues, consistent with the quantitative differences amongst the PIs in their activity (Figure 4C). The HGP of fourth-instar larvae displayed at least seven protease isoforms (HGP-1 to −7), of which only HGP-6 and −7 were able to retain marginal activity in the presence of CanPIs from either uninduced or induced tissues (Figure 4D).

**Figure 5:**
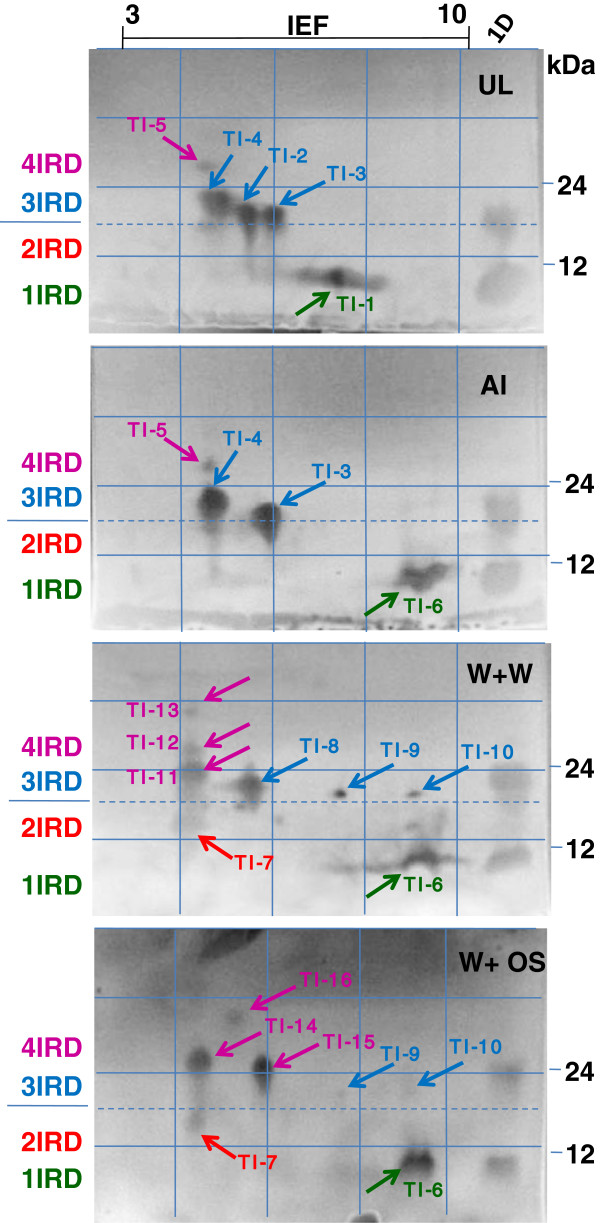
**2D-TI activity profiles of uninduced and induced *C. annuum *leaves (systemic tissues).** Equal TI units from leaf extracts were visualized by GXCT after separation by 2-D as described in Materials and Methods. Differential TI activity isoforms, displaying charge and mass variations, were observed in induced leaves. Grids are superimposed across the gel images to help visualize the major shifts in the molecular mass and pI of the induced TI isoforms. The increased appearance of TI isoforms (TI-7 to −16) is evident and they correspond to 3- and 4-IRD CanPIs. Similar activity profiles were observed for local tissues of induction treatments.

The 2-D activity profiles of induced leaf samples showed the induction of several novel PI isoforms in the range of isoelectric point (pI) from 4 to 7 and also shift in the pI and/or molecular mass of few isoforms (Figure 5). Five prominent TI isoforms (TI-1 to TI-5) corresponding to 1-, 2-, 3- and 4-IRD were identified in uninduced leaves, in the pI range of 5 to 7 (Figure 5). TI-6 (1-IRD) isoform which has major basic shift in pI compared to TI-1, was present in all the three types of induced tissues. TI-3, -4 and −5 were consistently detected in aphid-infested leaves but were absent from wounded leaves treated with water or OS. TI-8 to −13 were present only in wounded leaves treated with water, while TI-14, -15, and −16, corresponding to 4-IRD CanPIs, were present in wounded leaves treated with OS only. The induced PI activity in wounded leaves treated with water showed a distribution of several TI isoforms with low intensity, whereas the up-regulation of only a few TI isoforms was evident in wounded leaves treated with OS. TI-2 was absent in all induced tissues, consistent with the specificity of some PI isoforms in uninduced leaves. Among the TI isoforms corresponding to 1-, 2-, 3- or 4-IRD CanPIs, more variations were observed for 3-and 4-IRD CanPIs.

**Figure 6:**
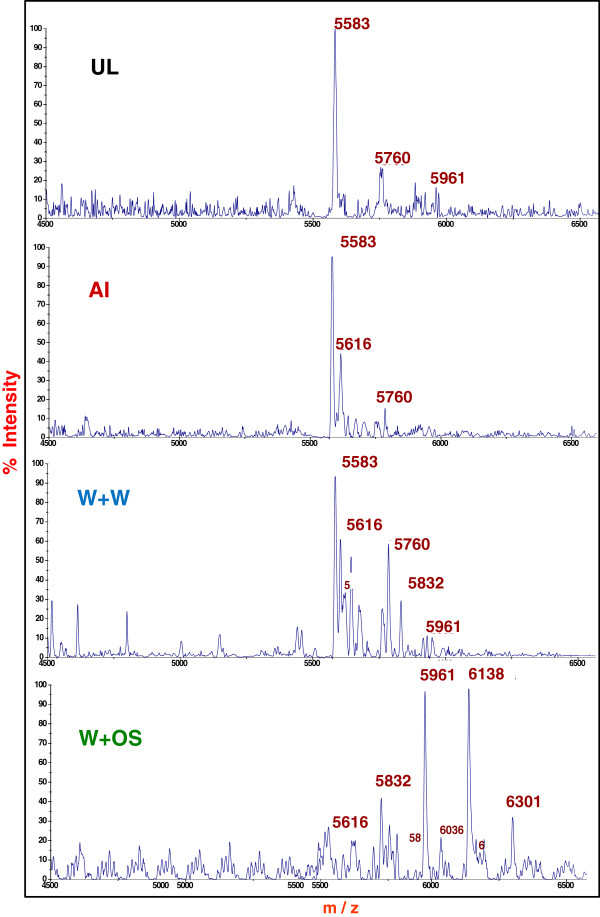
**MALDI-TOF-MS characterization of partially purified PIs from leaf extracts (systemic tissue in case of inductions).** Mass spectral analysis revealed peaks of varying masses in the range of 5.5 to 6.3 kDa across treatments. These represent an1-IRD peptide, which was further confirmed by MS/MS analysis. The increased diversity of processed IRDs is prominent in *C. annuum* leaf tissues wounded and treated with water (W+W) or oral secretions (W + OS) as compared to aphid infested.

Partially purified PIs contained small peptides of about 5.5 to 6.3 kDa equivalent to a single IRD as analyzed on MALDI-TOF-MS (Figure 6). High molecular mass proteins exhibited very low intensity in the mass spectra, perhaps due to ion suppression effects, and therefore are not considered. Uninduced leaf extracts displayed a single major peak of 5583 Da, whereas aphid-infested leaves had major peaks at 5583 Da and 5616 Da and few low intensity peaks. A peak of 5583 Da was most prominent in wounded leaves treated with water, in addition to several peaks of 5616, 5760 and 5832 Da with high intensity. Extract from leaves wounded and treated with OS displayed 6138 and 5961 Da as the major peaks, with low intensity peaks at 5616, 5832, 6036 and 6301 Da and an absence of the 5583 Da peak. This variation in the molecular masses of peptides is likely a result of the proteolytic processing of the precursor PI proteins to generate multiple functional PI species (equivalent to single IRD from multi-IRD CanPIs) [22,31,32].

The partially purified PI protein from uninduced and induced leaves, displaying varying mass spectral profiles on MALDI-TOF-MS, were separated on Tricine gel, and the proteins were individually excised and processed for in-gel digestion followed by the identification of peptides by MALDI-TOF-MS/MS (Additional file 1: Table S2). The 6 kDa protein in all the extracts showed matches to Pin-II proteinase inhibitors from *C. annuum*. However, due to high homology amongst IRD sequences, the database generated identification to several CanPIs/IRDs (Additional file 1: Table S2).

### Tissue specificity of PI accumulation

**Figure 7:**
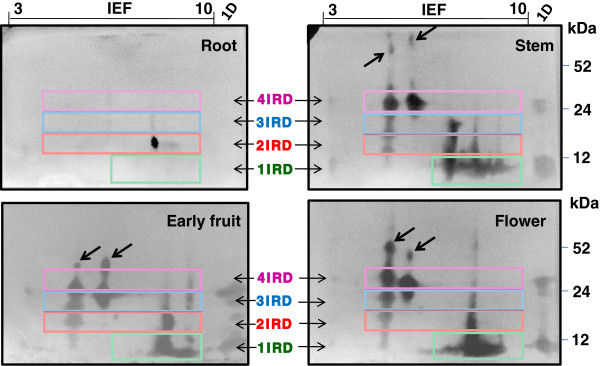
**2D-TI activity profiles of various *C. annuum *plant parts under uninduced conditions.** Several TI activity isoforms were detected in all the tissues except root tissue, which shows very low TI activity and only one TI isoform. Qualitative and quantitative variations in TI profiles of tissue from stem, early fruit and flower are quite evident and the differences are highlighted through the clusters of 1-, 2-, 3- and 4-IRD PIs.

Flowers, followed by stems and early fruit showed a significantly higher level of PI activity compared to tissue from leaves, roots and the different developmental stages of fruit (Additional file 4: Figure S3). Flower tissue showed the highest PI activity, while tissue from turning fruit exhibited the least activity with a 7-fold difference. The in-gel PI visualization after resolution on 2-DE for various *C. annuum* plant parts displayed the qualitative variations in the PI activity across these tissues (Figure 7). The clusters corresponding to 1-, 2-, 3- and 4-IRD CanPIs were prominent in stems, early fruit and flowers. In accordance to the very low TIUs in roots, we could detect only one TI isoform which corresponded to 2-IRD CanPI. The 1-IRD cluster contained several merged TI isoforms and indicated multiple, charge variants due to amino acid sequence differences. Stem tissue in particular showed less diversity in the 3-IRD cluster while exhibiting the highest number of charge variants in the 1-IRD cluster. Stem, early fruit stage and flower tissues revealed the presence of the high molecular mass isoforms of PIs (Figure 7, marked by arrows) that are predicted to be larger than those of 4-IRD CanPIs.

## Discussion

*C. annuum* produces an array of PI genes exhibiting regulated expression under induced conditions. CanPIs are strongly elicited by wounding and upon Lepidopteran insect attack owing to the high cellular damage and plant’s perception of herbivore-specific elicitors as compared to aphid infestation. Based on observed *CanPI* accumulation upon elicitation, it is intriguing to speculate that generating sequence diversity in the form of multi-IRD PIs is part of a plant’s defense strategy to obtain a pool of diverse functional units to confine insect attack.

Among Solanaceae, different species of *Nicotiana* display PI genes containing 2 and 4 to 8 IRDs [19]. In addition, 2-IRD PIs from tomato (*Solanum lycopersicum)* and *S. nigrum* have been well characterized [4,33]. Simultaneously expressed PIs with varying IRD composition have been reported in *N. alata* stigma (4- and 6-IRDs; [34]), in *N. glutinosa* infected with TMV (6- and 8-IRD; [35]) and in *N. attenuata* in response to herbivory (7-IRD; [22]). In addition to the previously reported 23 PI genes from *C. annuum* pericarp, developing fruit and stem [16,26,27], 24 new *CanPIs* were isolated and characterized from the induced leaves in the present study. Among these 47 *CanPI* genes, 9 contained 4-IRDs, 20 contained 3-IRDs, 15 contained 2-IRDs and 3 contained a single IRD thus contributing to the diversity. Consistent with the previous studies, 3-IRD PIs were highly abundant in *C. annuum* leaves (Table 1, Figure 2). We observed a strong up-regulation of 3- and 4-IRD *CanPI* transcripts in induced leaves when compared to uninduced leaves (Figure 1A, Table 1). Reciprocating patterns obtained at the PI activity level corroborate the induction specific regulation of CanPIs. Other reports on *C. annuum* have also demonstrated an increase in *CanPI* expression in local and systemic leaf tissues upon elicitation by aphids, viruses, insect feeding and mechanical wounding [16,25,26]. The stronger induction of PI activity in distant leaves than in local leaves challenged with the same treatment was interesting and suggested strong distant signalling.

Despite the difficulties of directly comparing the aphid infestation treatment with other treatments carried out in the present study, an interesting pattern of accumulated *CanPIs* is evident at transcript and PI activity level. *CanPI* transcript abundance in aphid infested leaves was much lower than that observed in wounded leaves treated with water or OS (Figure 1A). Aphids have been found to elicit defense related genes including PIs but the responses are low as compared to that elicited by chewing insect attack [36,37]. However, studies have also found aphid induced transcriptional signatures of salicylic acid signaling and no increase in PI transcripts [38]. In the present study, transcripts of *CanPI-41* and −*43* are highly accumulated under aphid infestation while *CanPI-8* and −*11* remain either un-elicited or suppressed by aphid damage; the latter being up-regulated by wounding or W+OS treatment. The suppression of selected wound induced responses, rather than the lack of cellular damage, might also be responsible for the low responses to aphid attack [36]. Aphid infestation induced CanPI activity though much less as compared to wounded leaves treated with water or OS (Figure 4A). A unique aphid induced CanPI signature was evident in the 2D activity profiles (Figure 5).

Particularly, 4-IRD PIs were strongly induced in wounded leaves treated with OS (Figure 1B) highlighting the strong and specific effects of insect elicitors on CanPI regulation. Plant responses to wounding/insect feeding are known to be specifically altered by the plant’s perception of herbivore-specific elicitors [8,39-42]. High amounts of jasmonic acid and the rapid accumulation of wound-inducible transcripts have been reported in response to insect damage or insect OS when compared to only mechanical wounding [5,40,43]. Wounded leaves treated with water showed the highest number (10) of uniquely expressed *CanPIs* (Figure 1C), though in lower frequency as compared to a few CanPIs (−4, -7, -10) and IRDs (4, 5, 10, 14, 25) with higher frequency in wounded leaves treated with OS. Specifically high representation of *CanPIs* with multiple IRDs directed towards enriching the PI blend with both CI and TI activities seems to be an approach adapted by the plant upon Lepidopteran insect attack, helpful in tackling a wide range of insect proteases [31]. Significantly high PI activity (Figure 4A) and detection of three PI activity bands in wounded leaves treated with water or OS in comparison to two activity bands in uninduced and aphid infested leaves (Figure 4B) is indicative of quantitative and qualitative variations in the accumulated PI activity. Further characterization by 2D electrophoresis, revealed the presence of multiple charge and/or molecular mass variants observed in wounded leaves treated with water (Figure 5; TI-7 to −13) and with OS (Figure 5; TI-14 to TI-16) clearly indicating the induced isoform diversity. The differential isoforms detected mostly corresponded to 3- and 4-IRD PIs and thus correlates with the high *CanPI* transcript accumulation under these two treatments. The absence of certain TI isoforms in OS treated leaves compared to wounding alone, suggests the suppression of some induced responses, resulting in treatment specific patterns. With respect to HGP inhibition potential, the PI activity in all leaf tissues attained 70% inhibition of HGP (Figure 4C) and could inhibit almost all the HGP isoforms (Figure 4D). However, an early saturation of HGP inhibition by proteins from leaves induced by wounding and/or treated with OS, as compared to uninduced leaves, is suggestive of the high quantitative accumulation of PI units in such leaf tissues and higher specific activity against insect gut proteases. Multiple IRDs are known to be generated from precursor *N. alata* PI proteins in Me-JA-elicited leaves [22,44] and from CanPI precursor proteins by the action of endogenous proteases at the linker regions [16]. An increase in the number and intensity of variant mass peaks equivalent to single IRDs, in wounding with water and with OS protein fractions (Figure 6), suggests the enhanced proteolytic processing of the up-regulated CanPI precursor proteins [22,31,32]. The specific presence of isoform TI-6 and absence of TI-1 (Figure 5) under all inductions also indicate the accumulation of differential 1-IRD isoforms that are generated as a result of processing of CanPI precursors. Thus, our results substantiate the hypothesis [22] that elicitation leads to over-production of the CanPI precursors and enhanced, differential processing of the precursors by proteases to IRDs, resulting in structurally and functionally diverse processed products. It was also noticed that induction treatment specificity is maintained even at the level at which precursor proteins are processed. Peaks ranging from 5.9 to 6.3 kDa show high intensity in wounding with OS treatment whereas the peaks from 5.5 to 5.8 kDa are prominent in uninduced and other treatments, aphid infested and wounding treated with water. These results affirm that plants can differentially perceive various kinds of biotic stresses and respond appropriately through regulation of PIs at transcriptional and post-translational levels.

Sequence analysis revealed highly homologous *CanPIs* with an average variance of 4%. The clear absence of partial N- and C-terminal repeats in the *CanPI* precursors groups them as distinct from *N. benthamiana* Pin-II PIs (Figure 2). The explicit clustering of *C. annuum* PIs from all other Solanaceous Pin-II PIs suggests recent evolutionary origins [21]. The diversity in CanPIs can be attributed to individual IRDs, which display a sequence variation ranging from 2 to 20% within the vicinity of the reactive site loops and C-terminal region. Twenty eight unique IRDs, constituting 7 CIs and 21 TIs, follow the H-L type topology, where the sequence repeat is identical to the structural repeat [21]. The induction-specific IRD distribution is predominantly biased towards TIs rather than CIs. It is known that in Lepidopteran insects, trypsin-like proteases are predominant which could be correlated to the relatively high abundance of trypsin specific PIs in plants.

Active site variants of TIs ‘CPRNC’, ‘CPKNC’, ‘CPRYC’ and ‘CPRDC’ and two types of CI sites ‘CTLNC’ and ‘CTPNC’ were present among all identified 28 IRDs. Interestingly four cysteine variants either, missing one or more conserved cysteine residues, change in position of cysteines or having additional cysteines were identified in the present study (Figure 3A). Recently, six natural IRD variants with selective losses of cysteine residues have been identified in potato [45]. The loss of cysteine residues is often associated with functional differentiation and suggests positive evolutionary gene selection. However, the nature of mutations and the associated factors responsible for selective losses of cysteines remains unclear. Studies on the significance of such mutations affecting the proteinase binding affinity and structural stability/integrity of IRDs have initiated an active debate on the evolution of disulfide bonds in the Pin-II family ( [45,46], our unpublished results). Particularly in *C. annuum*, the identification of four such IRDs as a result of various inductions suggests that plants elaborative defenses by expressing modified IRDs that improve their overall activity against target proteases. Among various tissues from *C. annuum*, flowers revealed the highest accumulation of PI activity (Additional file 4: Figure S3), consistent with a role in protecting the reproductive parts of the plant against pests as reported in tomato [15,44].

## Conclusions

This study suggests that CanPI sequence diversity, tissue specificity and explicit responses to different inductions are part of effective plant defense system. The significance of huge complexity of PIs observed specifically in *C. annuum* needs to be understood. Recent reports on the endogenous and/or defensive roles of PIs from various Solanaceous species and simultaneous expression of multiple *CanPIs* constitutively highlight their prospective involvement in many of the plant’s complex processes [16,19,23,47-49]. Moreover, up-regulated yet specialized *CanPI* expression upon wounding and insect infestation provides insights into the evolution of PI based plant defense mechanisms against insects and generates many unanswered questions about their regulation. Essentially, more functional studies need to be performed for specific *CanPI* genes in order to ascertain their roles under a particular treatment and how this variation accounts for the fitness benefit of the plant under specific biotic stress conditions.

## Methods

### Plant material and induction treatments

*C. annuum* seeds (cv PhuleJyoti) (diploid) were grown in pots with Soilrite (Mixture of horticulture grade expanded perlite, Irish Peat moss and exfoliated vermiculite in equal ratio; Naik Krushi, Pune, MS, India) and supplemented daily with Hoagland solution. 30-day-old seedlings were transferred to individual pots and grown in a growth chamber maintained at 23°C (±2°C) with a 14 h light photoperiod. Leaves, stems, various stages of fruits (early, mid, turning and late), roots and flowers from mature plants (3 months old) were harvested for screening tissue-specific CanPIs.

All induction experiments were performed on 3-month-old plants and a set of 3 plants was taken per treatment for each biological replicate. Leaves were mechanically wounded by rolling a fabric pattern wheel along the length of the lamina (4 to 6 rolls depending on the size of leaf) and the resulting puncture wounds were immediately treated with water or OS. These were considered local tissue, whereas the non-wounded leaves one node above or below were harvested to measure systemic responses. Local and systemic tissues were collected after 30 h of treatment. OS used was collected from *H. armigera* larvae and diluted 1:50 (v/v) times in MQ water. Plants were kept in an open garden for passive aphid infestation. Natural infestation by *Myzus persicae* was observed on *C. annuum* leaves within a week. The density of aphids was high on the leaf lamina towards the petiole. Leaves with at least 20 nymphs per leaf growing at the same nodes were collected as local tissue whereas non-infested leaves were harvested as systemic tissues. Since the plants were naturally infested by aphids in open conditions, the possible comparisons with this treatment and the other two, which were performed in a highly specific and controlled manner, are limited. Leaves from un-elicited control plants growing at the same nodes were harvested from uninduced plants. All the tissue collections were done at a same time and were flash-frozen in liquid nitrogen and stored at -80°C until further use. Two biological replicates were used for the whole study.

### Expression profiling, cloning of Pin-II genes and sequence analysis

Total RNA from *C. annuum* leaf tissues (uninduced and all three inductions-systemic) was isolated using TRIZOL (Invitrogen, Carlsbad, CA, USA) followed by DNAse treatment at 37°C. Purified RNA was quantified by spectrophotometry, and 1.5 μg was used for first-strand cDNA synthesis using a reverse transcriptase kit (Promega, Madison, WI, USA). Proof-reading Accuprime Pfx DNA polymerase (Invitrogen) was used to amplify cDNAs from systemic leaf tissues of individual treatments and uninduced leaf in independent PCR reactions using *CanPI* gene (Genbank accession: AF039398) specific primer pair (CanPin-1F and CanPin-1R; Additional file 1 Table S1A). Amplicons were cloned into pGEMT-easy vector (Promega). 60 cloned fragments from each treatment were sequenced using standard T7 forward and SP6 reverse primers. Sequence editing and analysis was carried out using BioEdit, Clustal-X and Lasergene software. For semi-quantitative analysis of *CanPIs* within the tissues, independent PCRs were performed with reduced number of cycles (25), Accuprime Pfx DNA polymerase and *CanPI* gene specific primer pair. Specific primer pairs were designed for individual *CanPI* genes in order to check the expression of specific *CanPIs.* However, due to a high degree of similarity/homology within *C. annuum PIs*, it was possible to design gene-specific primer pairs for *CanPI-3*, -*5*, *-7*, *-8* and −*10* only. The specific pairs of oligonucleotides used for the internal differentiation of individual *CanPIs* are stated in Additional file 1: Table S1A and B. All PCRs were carried out in technical duplicates.

### Protein extraction and proteinase inhibitor activity assays

Total soluble protein was extracted from 1g of fresh leaf tissue obtained from uninduced and induced (local and systemic for all treatments) *C. annuum*, using a mixture of water and 5% polyvinylpolypyrrolidone (Sigma, St. Louis, MO, USA)*.* Following protein estimation using Bradford reagent (Bio-Rad Laboratories, Hercules, CA, USA), the trypsin inhibitory activity and *H. armigera* gut protease (HGP) inhibitory activity was estimated enzymatically in the leaf extracts using the synthetic substrate Benzoyl-DL-Arginyl-*p*-Nitroanilide (Sigma, St. Louis, MO, USA) as described earlier [28]. Protease inhibitor activity was expressed as trypsin inhibitory units per mg of tissue (TIUs/mg). Various amounts of the leaf protein extracts were titrated against HGP to determine maximum inhibitory activity. All the assays were carried out in technical triplicates with 2 biological replicates and statistical analysis of data was performed using single-factor ANOVA followed by Tukey’s post hoc analysis.

### 1D and 2D electrophoresis for in-gel identification of proteinase inhibitory activity

Equal TIUs of leaf protein extracts were resolved on 12% native-PAGE and further processed in order to visualize trypsin inhibitor isoforms using the previously described gel X-ray film contact print (GXCT) method [50]. To visualize protease activity, protein extracts incubated with HGP were resolved on 8% native-PAGE gel and processed by GXCT [51]. For 2D electrophoresis, acetone-precipitated proteins were re-suspended in rehydration buffer and separated in first dimension (Isoelectric focusing) using 11-cm IPG strips (pH 3–10 NL; Bio-Rad Laboratories, Hercules, CA, USA) as per the manufacturer’s protocol. Second-dimension separation was done on 12% SDS-PAGE gel using a Hoefer electrophoresis unit (GE Healthcare Bio-sciences AB, Buckinghamshire, UK) maintained at 24°C and 200 V. Trypsin inhibitor activity was visualized by modified GXCT [52]. All the electrophoresis experiments were carried out in technical triplicates with 2 biological replicates.

### Partial purification and proteomic analysis of leaf protein extracts

To identify the proteinase inhibitors from *C. annuum* leaf tissues, protein extracts were first partially purified in the following manner. Ammonium-sulphate-precipitated proteins (90% saturation) from leaf extracts were resuspended in 50 mM Tris buffer (pH 8.0), and subsequently treated at 65°C for 10 min before being desalted using PD SpinTrap^TM^G-25 column (GE Healthcare). Proteins were then separated on DEAE-Sephacel (GE Healthcare) equilibrated with 50 mM Tris buffer (pH 8.0), and flow-through was collected separately from bound proteins that were eluted with a NaCl gradient of 0.25 M to 0.4 M in 50 mM Tris buffer (pH 8.0). Fractions with trypsin inhibitor activity were concentrated and desalted using a PD Spintrap column. The partially purified protein fractions were pooled and qualitatively analyzed by matrix-assisted laser desorption ionization time-of-flight mass spectrometry (MALDI-TOF-MS), Voyager-De-STR (Applied Biosystems, Framingham, MA, USA). Mass spectral acquisition was performed with a standard instrumental protocol as described earlier [31]. In brief, 2 μg of protein sample was mixed with 20 μL of freshly prepared sinapinic acid (Sigma-Aldrich) (in 30% acetonitrile [ACN], 0.1% trifluoroacetic acid [TFA]) and spotted on the stainless steel MALDI plate, and spectral profiles were acquired in the range of 1 to 25 kDa. The spectra were analyzed with Data Explorer™ for regions of interest and processed for advanced base-line correction and noise removal.

To obtain protein sequence data, the partially purified proteins were separated on 16% Tricine SDS-PAGE gel [53] to resolve the low molecular mass proteins, after which protein bands were in-gel digested and analyzed by MALDI-Q-TOF- MS (SYNAPT High Definition Mass spectrometer, Waters Corporation, Milford, MA, USA). Mass spectral acquisition was carried out by MALDI survey method. Protein Lynx Global server version 2.4 software (Waters) was used for data processing and database searches. The MS/MS data was searched against the Pin-II protein database constructed separately using the following parameters: peptide tolerance of 20 ppm, fragment tolerance of 0.05D, one missed cleavage, carbamidomethylation of cysteines and possible oxidation of Methionine.

## Abbreviations

Aa: Amino acid; CI: Chymotrypsin inhibition; HGPI: *H. armigera* gut protease inhibition; MALDI-TOF-MS: Matrix-assisted laser desorption ionization time-of-flight mass spectrometry; IRDs: Inhibitory repeat domains; OS: Oral secretions; PI: Proteinase inhibitor; TI: Trypsin inhibition.

## Competing interests

The authors declare that they have no competing interests.

## Authors’ contribution

MM carried out the induction experiments, sequence analysis, characterization of PI protein activity and drafted the manuscript. NSM was involved in protein extractions and the 2D gel electrophoresis. VAT participated in design of the study, sequencing data analysis and helped to draft the manuscript. MJK helped in performing the MALDI-TOF-MS analysis and protein identifications. ITB and VSG helped in interpretation of results and drafting the final version of the manuscript. APG conceived the study and helped in drafting the manuscript. All authors contributed at the draft stage of writing and all approved the final manuscript.

## Supplementary Material

Additional file 1 **Table S1.** Oligonucleotide primers used for RT-PCR and *CanPI* internal differentiation. **Table S2:** Protein identification by MALDI-TOF-MS/MS, database searches. Click here for file

Additional file 2 **Figure S1.** Multiple sequence alignment of deduced aa sequences of signal peptides (SP-1 to SP-10) of *CanPI* genes, displaying variations. Click here for file

Additional file 3 **Figure S2.** Multiple sequence alignment of deduced aa sequences of IRDs (28 in number) constituting all the *CanPI* genes. The reactive site residue P1 is marked by an arrow. Presence of Lys (K) or Arg (R) at P1 site, indicates trypsin inhibitory site (TI) and Leu (L) indicates chymotrypsin inhibitory site (CI). The core reactive site is marked by an orange box. Click here for file

Additional file 4 **Figure S3.** Tissue-specific TI activity in various tissues of a mature *C. annuum* plant. Concentration is represented in terms of trypsin inhibitory units (TIUs/mg). Flower tissue shows the highest TIUs with an almost 7-fold increase compared to leaf tissue. Stem and early fruit tissue also shows significantly higher TI activity. Click here for file
