# Supplementary material for: Stress inducible proteinase inhibitor diversity in Capsicum annuum
Source: BMC Plant Biol. 2012 Nov 16;12:217. doi: 10.1186/1471-2229-12-217 (PMC3511207; doi:10.1186/1471-2229-12-217)
Supplement: Additional file 1 — Table S1. Oligonucleotide primers used for RT-PCR and CanPI internal differentiation. Table S2: Protein identification by MALDI-TOF-MS/MS, database searches. [file 1471-2229-12-217-S1.docx]

**Supplementary Table 1:**

**[A]: Oligonucleotide sequences of primers used for RT-PCR and CanPI internal differentiation**

| **Primer name** | **Primer sequence** | **Region** |
| --- | --- | --- |
| Can 18s rRNA F | 5’ CCG GTC CGC CTA TGG TGT GCA CCG G 3’ | - |
| Can 18s rRNA R | 5’ CCT CTG ACT ATG AAA TAC GAA TGC CCC 3’ | - |
| CanPin-1F | 5’ ATG GCT GTT CCC AAA GAA G 3’ | 5’ end- Signal Peptide (SP) |
| CanPin-1R | 5’ CTG TTC ATG CTT TTA CTT TTC 3’ | 3’ end of CanPI gene |
| V49-F | 5’ GCT TCC CTA CTT GTA CTT GG 3’ | Internal (within SP) |
| V51-R | 5’ CAA TTG GTG CAT ATG GGT C 3’ | Internal- towards 3’ end |
| V52-R | 5’ C ACA GTT CAG AGT GCA AGC 3’ | Internal- towards 3’ end |
| V53-R | 5’ T CTT GGA TCA CAG TTC AGA GTG 3’ | Internal- towards 3’ end |
| V57-F | 5’ GCC TTC CTA CTT GTT CTT G 3’ | Internal (within SP) |
| V58-F | 5’ GCT TCC TAC TTG TTC TTG G 3’ | Internal (within SP) |
| V63-F | 5’ CAA AGA AAC GCA AAA GAA CC 3’ | Internal (within linker) |

**[B]: Oligonucleotide pairs for internal differentiation of CanPIs**

| **Primer pair** | **Specific CanPI** | **Amplicon size (bp)** |
| --- | --- | --- |
| CanPin-1F, CanPin-1R | All CanPIs | 789, 614, 455, 267 |
| V57, CanPin-1R | CanPI-3 | 600 |
| V58, CanPin-1R | CanPI-5 | 585 |
| CanPin-1F, V51 | CanPI-10 | 644 |
| V63, V53 | CanPI-7 | 552 |
| V49, V52 | CanPI-8 | 574 |

**Supplementary Table 2: Protein identification by MALDI-TOF-MS/MS, database searches.**

| **Tissue** | **Accession No.** | **Identified protein** | **Protein identification data** | | |
| --- | --- | --- | --- | --- | --- |
|  |  |  | **(No. Pep./coverage %)** | **PLGS Score** | **Peptide sequence (MS/MS)** |
| **Uninduced** | Q4ZIQ3 | Pin II type proteinase inhibitor 9 OS Capsicum annuum   \| Pin-II PI 21, 17, 20, 13, 23, 6, 4, 3,  5, 16, 12, 14 \| \| --- \| | 15/ 45 | 2601 | \| (R)KGCNYYSADGTFICEGESDPNNPK(P) \| \| --- \| \| (K)GCNYYSADGTFICEGESDPNNPK(P) \| \| (R)LCTNCCAGR(K) \| \| (R)KGCNYYSADGTFICEGESDPNNPKPCTLNCDPR(I) \| \| (K)GCNYYSADGTFICEGESDPNNPKPCTLNCDPR(I) \| \| (R)KGCNYYSADGTFICEGESDPNN(P) \| \| (R)KGCNYYSADGTFICEGESDPNNPK(P) \| \| (K)GCNYYSADGTFICEGESDPNNPK(P) \| \| (A)DGTFICEGESDPNNPK(P) \| \| (I)CEGESDPNNPK(P) \| \| (D)PNNPK(P) \| \| (R)LCTNCCAGR(K) \| \| (L)CTNCCAGR(K) \| \| (C)TNCCAGR(K) \| \| (R)KGCNYYSADGTFICEGESD(P) \| |
| **Aphid infested**  **(systemic tissue)** | Q4Z8K3 | Pin II type proteinase inhibitor 3 OS Capsicum annuum  Pin-II PI 6, 21, 20, 13, 23, 4, 17, 9, 5, 16, 19, 14, 12 | 18/ 51 | 718 | \| (N)RICTNCCAGR(K) \| \| --- \| \| (R)KGCNYYSADGTFICEGESDPNNPK(A) \| \| (R)LCTNCCAGR(K) \| \| (K)GCNYYSADGTFICEGESDPNNPK(A) \| \| (R)SEGNAENRICTNCCAGR(K) \| \| (K)VSFLAFLLVLEILLLHVDAKACSEENAENR(I) \| \| (R)NCDPNIAYSLCLYEK(-) \| \| (N)RICT(N) \| \| (N)RICTN(C) \| \| (N)RICTNC(C) \| \| (T)NCCAGR(K) \| \| (N)CCAGR(K) \| \| (C)CAGR(K) \| \| (Y)YSADGTFICEGESDPNNPK(A) \| \| (R)LCTNCCAGR(K) \| \| (L)CTNCCAGR(K) \| \| (C)TNCCAGR(K) \| \| (N)RIC(T) \| |
| **Wounding + water**  **(systemic tissue)** | D2CGT4 | Pin II type proteinase inhibitor 17 OS Capsicum annuum  Pin-II PI 6, 21, 20, 13, 23, 4, 3, 9, 5, 16, 19, 8, 14, 12 | 17/ 60 | 718 | \| (N)RICTNCCAGR(K) \| \| --- \| \| (R)KGCNYYSADGTFICEGESDPNNPK(A) \| \| (R)LCTNCCAGR(K) \| \| (K)GCNYYSADGTFICEGESDPNNPK(A) \| \| (K)VSFLAFLLVLEILLLHVDAKACSEENAENR(I) \| \| (R)NCDPNIAYSLCLYEK(-) \| \| (N)RICT(N) \| \| (N)RICTN(C) \| \| (N)RICTNC(C) \| \| (T)NCCAGR(K) \| \| (N)CCAGR(K) \| \| (C)CAGR(K) \| \| (Y)YSADGTFICEGESDPNNPK(A) \| \| (R)LCTNCCAGR(K) \| \| (L)CTNCCAGR(K) \| \| (C)TNCCAGR(K) \| \| (N)RIC(T) \| |
| **Wounding + OS**  **(systemic tissue)** | D2CGT4 | Pin II type proteinase inhibitor 17 OS Capsicum annuum  Pin-II PI 21, 19, 20, 23, 13, 6, 4, 3, 9, 5, 16, 12, 8, 14 | 12/ 29 | 603 | \| (R)KGCNYYSADGTFICEGESDPNNPK(A) \| \| --- \| \| (K)GCNYYSADGTFICEGESDPNNPK(A) \| \| (R)LCTNCCAGR(K) \| \| (N)RICTNCCAGR(K) \| \| (N)RICTN(C) \| \| (N)RICTNC(C) \| \| (N)CCAGR(K) \| \| (C)CAGR(K) \| \| (R)KGCNYYSADGTFICEGESDPNN(P) \| \| (R)LCTNCCAGR(K) \| \| (C)TNCCAGR(K) \| \| (N)RIC(T) \| |
