# Supplementary figures and images for: Stress inducible proteinase inhibitor diversity in Capsicum annuum
Source: BMC Plant Biol. 2012 Nov 16;12:217. doi: 10.1186/1471-2229-12-217 (PMC3511207; doi:10.1186/1471-2229-12-217)

## Slide 1
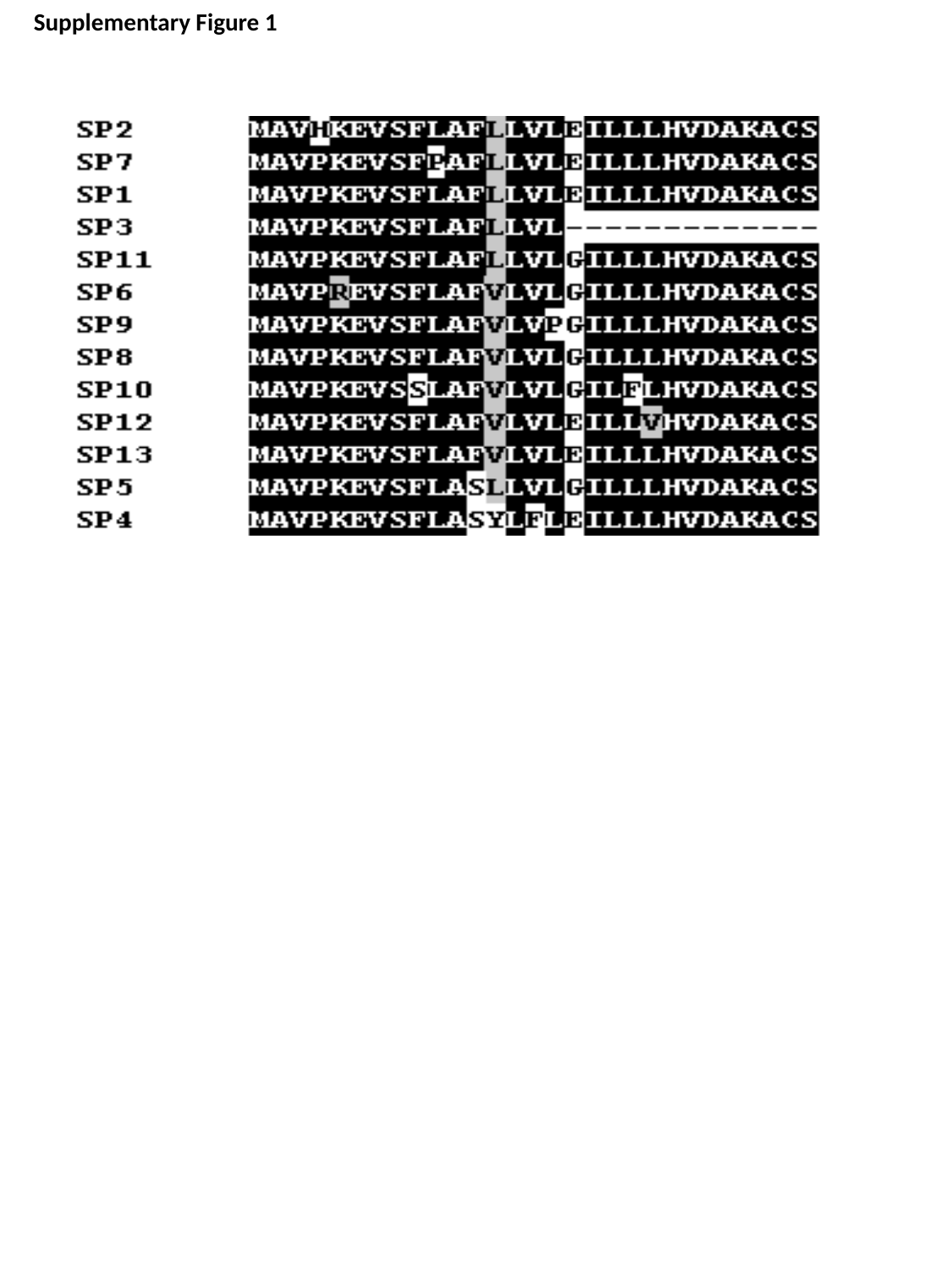

Supplementary Figure 1

Supplement: Additional file 2 — Figure S1. Multiple sequence alignment of deduced aa sequences of signal peptides (SP-1 to SP-10) of CanPI genes, displaying variations. [file 1471-2229-12-217-S2.pptx]
