# Supplementary material for: Stress inducible proteinase inhibitor diversity in Capsicum annuum
Source: BMC Plant Biol. 2012 Nov 16;12:217. doi: 10.1186/1471-2229-12-217 (PMC3511207; doi:10.1186/1471-2229-12-217)
Supplement: Additional file 3 — Figure S2. Multiple sequence alignment of deduced aa sequences of IRDs (28 in number) constituting all the CanPI genes. The reactive site residue P1 is marked by an arrow. Presence of Lys (K) or Arg (R) at P1 site, indicates trypsin inhibitory site (TI) and Leu (L) indicates chymotrypsin inhibitory site (CI). The core reactive site is marked by an orange box. [file 1471-2229-12-217-S3.pptx]

## Slide 1
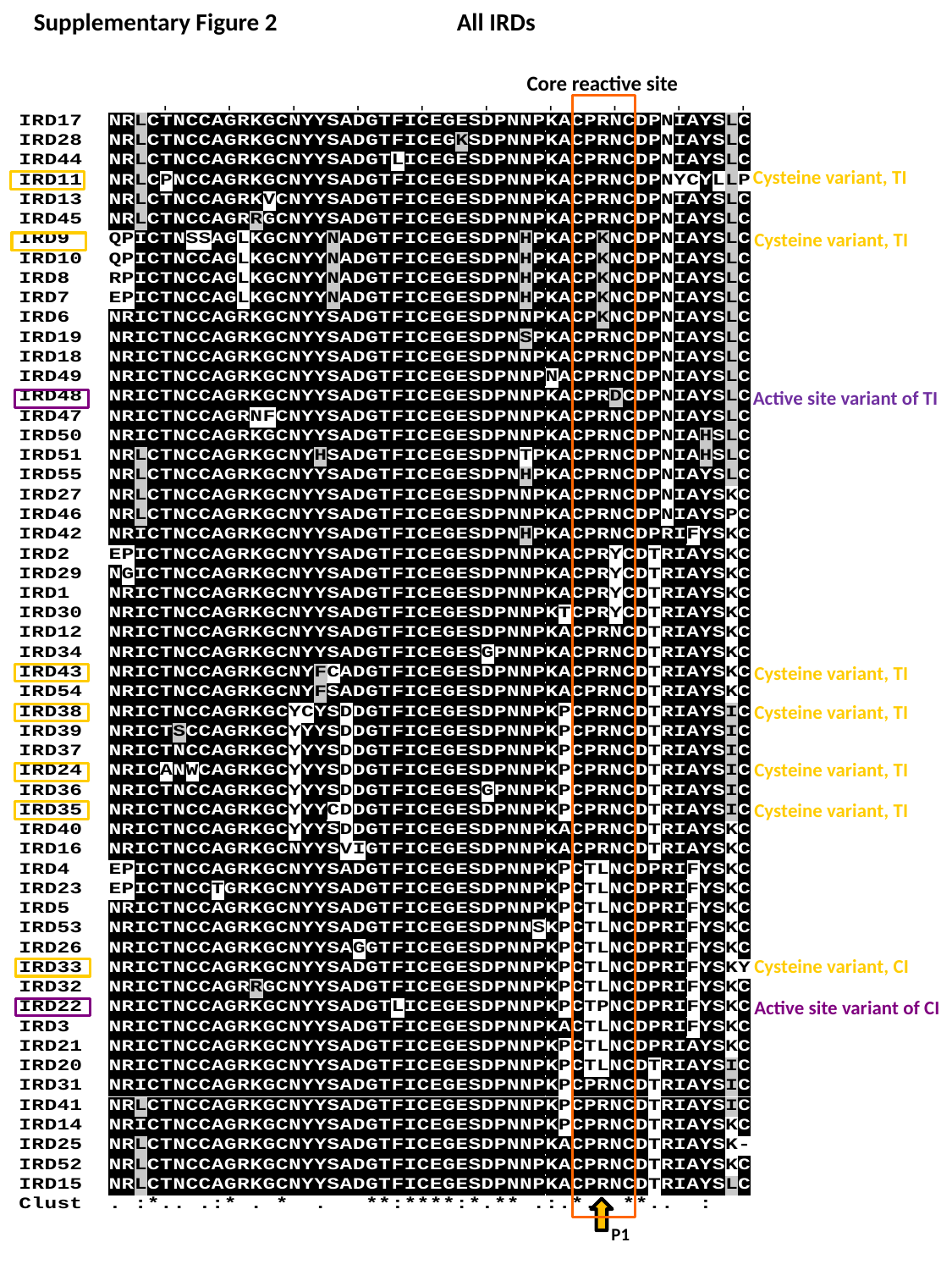

Supplementary Figure 2
All IRDs
Core reactive site
Cysteine variant, TI
Cysteine variant, TI
Active site variant of TI
Cysteine variant, TI
Cysteine variant, TI
Cysteine variant, TI
Cysteine variant, TI
Cysteine variant, CI
Active site variant of CI
P1
