# Supplementary material for: Stress inducible proteinase inhibitor diversity in Capsicum annuum
Source: BMC Plant Biol. 2012 Nov 16;12:217. doi: 10.1186/1471-2229-12-217 (PMC3511207; doi:10.1186/1471-2229-12-217)
Supplement: Additional file 4 — Figure S3. Tissue-specific TI activity in various tissues of a mature C. annuum plant. Concentration is represented in terms of trypsin inhibitory units (TIUs/mg). Flower tissue shows the highest TIUs with an almost 7-fold increase compared to leaf tissue. Stem and early fruit tissue also shows significantly higher TI activity. [file 1471-2229-12-217-S4.pptx]

## Slide 1
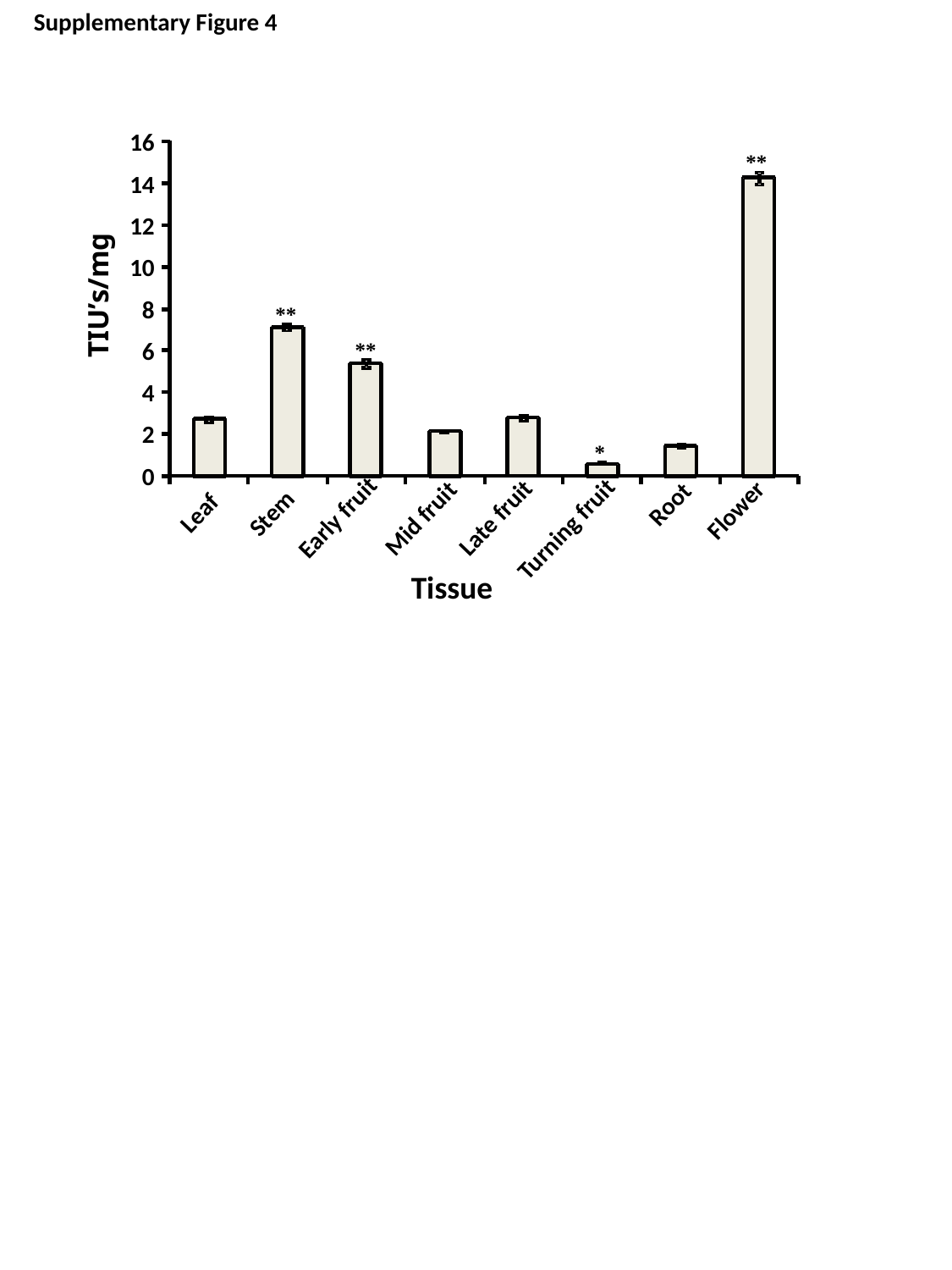

Supplementary Figure 4
16
**
14
12
TIU’s/mg
10
8
**
**
6
4
2
*
0
Root
Flower
Leaf
Stem
Early fruit
Late fruit
Mid fruit
Turning fruit
Tissue
